# Comparative Analysis of Tri-Polar Concentric Ring and Conventional Electrodes for Overt and Covert Speech

**DOI:** 10.3390/s26134084

**Published:** 2026-06-27

**Authors:** Paras Qadir Memon, Chuck Anderson, Zeeshan Qadir Memon, Shoaib Memon, Adnan Qadir

**Affiliations:** 1 Department of Computer Science, Colorado State University, Fort Collins, CO 80523, USA; chuck.anderson@colostate.edu; 2 Oregon State University, Corvallis, OR 97331, USA; 3 University of Arkansas at Little Rock, Little Rock, AR 72204, USA; 4 Aga Khan University, Karachi 74800, Pakistan

**Keywords:** electroencephalography (EEG), brain–computer interface (BCI), overt and covert speech, concentric ring electrodes, surface Laplacian, machine learning, deep learning, neural signal classification, biomedical signal analysis, Biosensors

## Abstract

The Brain–Computer Interface (BCI) is a system that enables communication between the brain and external devices by translating brain activity into commands. Electroencephalography (EEG) is a commonly used modality for measuring brain activity. However, its low signal-to-noise ratio (SNR) and electrode reference problems lead to poor spatial resolution. As a result, EEG signals are often contaminated with physiological artifacts such as muscle movements. Therefore, this study used novel tripolar concentric ring electrodes (TCREs) to record brain signals related to overt and covert speech. Brain signals associated with overt and covert speech were recorded using TCRE and disc electrodes. Classification algorithms, including K-Nearest Neighbors (KNN), Fully Connected Neural Networks (FCNN), and Convolutional Neural Networks (CNN), were used to classify the TCRE and conventional EEG signals. The data were collected from 16 healthy participants, consisting of 10 males and 6 females. The experimental results demonstrate that TCREs provide superior performance compared to conventional disc electrodes. In addition, the 0.5–1.2s interval, corresponding to the peak stimulus window, exhibits a maximum power of 250μV. The average accuracy achieved during this peak epoch was 86.25%, whereas the remaining epoch shows an accuracy of 83.5% using TCREs.

## 1. Introduction

The Brain–Computer Interface (BCI) is a system that enables direct communication between the brain and external devices [1]. These systems enable individuals with motor impairments to perform everyday tasks and interact with the external environment [2]. A number of signals are used in BCI, such as electromyography (EMG) [3], functional magnetic resonance imaging (fMRI) [4], magnetoencephalography (MEG) [5], electroencephalography (EEG) [6], and electrocorticography (ECoG) [7]. Among these signals, EEG is one of the most popular modalities of BCI systems because it is non-invasive and does not require surgery to insert electrodes in the brain. In the last few decades, BCI research has explored a wide range of applications involving different parts of the human body, such as the elbows [8], wrists [9], legs [10], tongue [11], and upper limbs [12]. Recent studies in neural engineering have provided experimental platforms to study functional neural networks. For example, human brain organoids have been used to explore neural network activity with microelectrode arrays and calcium imaging [13]. In addition, brain–machine interface technology has been used to support motor rehabilitation, speech restoration, and assistive communication in patients with neurological impairments, while also underscoring the need for improved signal acquisition and decoding methods [14]. Limited research has been done on the overt (aloud) and covert (silent) behavior of the brain. Overt and covert brain signals are very critical to understand because they help people who suffer from aphasia or speech impairment injuries and are unable to communicate with the outside world [15].

### 1.1. Overt and Covert Decoding Using Conventional EEG

Recent studies have investigated speech decoding using conventional EEG signals and different machine learning and deep learning algorithms. Hossain et al. [16] investigated imagined speech classifying of 26 letters of the English alphabet and 10 digits. Data were collected from 20 healthy participants in real time while they imagined letters and digits. The classification accuracy reported in this study was 85.65% for letters and 83.65% for digits using BiLSTM. In this study, the EEG data were prepared with 75% overlap segmentation, which can increase the number of samples with strong similarity in the training and test datasets, potentially limiting the validity of this result.

Jiang et al. [17] worked on decoding overt and covert EEG signals. Data were recorded using conventional EEG electrodes. The EEG data were recorded from 57 right handed native English speaking adult males. During the covert experiment paradigm, participants were asked to imagine the word silently, whereas in the overt condition, they pronounce the word aloud. During the experiment, the same word was repeated five times. Their results show that the model achieved a classification accuracy of 34.7%. Furthermore, in this study, the data were recorded from conventional EEG electrodes, which inherently have low spatial resolution, low signal-to-noise ratio, and reference electrode problem [18]. Similarly, Milyani and Attar conducted a pilot study on inner speech decoding. Data were extracted from a publicly available EEG-fMRI dataset (four participants). The study compared the results of the EEGNet-based architecture with those of the spectro-temporal transformer. The transformer model outperformed EEGNet and achieved an accuracy of 82.4%, whereas EEGNet achieved approximately 36% accuracy. In addition, it was observed that socially meaningful words (wife, father, child) yield better results compared to numerical words (six, ten). This shows that emotionally meaningful words may give better brain signals [19].

In addition, Alharbi and Alotaibi [20] conducted research on the publicly available BCI2020 dataset for imagined speech, which is based on 15 participants. The five imagined words were used: “Hello", “Help me,” “Stop,” “Thank you,” and “Yes". Hybrid deep learning algorithms were used for the decoding of imagined speech using EEG signals. The results show that the 3DCNN-BiLSTM model achieved a maximum accuracy of 75.2% for imagined words, while the 3DCNN-stackLSTM model yielded a maximum accuracy of 44.7%. Correspondingly, Abdulghani et al. [21] worked on decoding imagined speech brain signals. Data were recorded from four healthy participants using an eight-channel EEG acquisition system.The participants were instructed to imagine these commands: up, down, left, right. The results show a generalization accuracy of 92.50% using LSTM recurrent neural network. This high accuracy may be the result of overfitting, as a limited dataset negatively affects deep learning performance due to overfitting [22]. These studies show the potential of modern classification methods to analyze speech related EEG.

### 1.2. TCRE-Based EEG Studies

Conventional electrode EEG signals are affected by volume conduction, limited spatial resolution, and reference electrode problem [23,24]. Tripolar concentric ring electrodes (TCREs) have been proposed as an alternative approach. TCREs improve spatial selectivity and reduce the influence of distant common activity and reference-related effects [18]. Based on the earlier studies on TCREs, Besio et al. [25] worked to improve the spatial selectivity of the EEG signal recorded from the brain. Six males and four females between the ages of 23 and 27 participated in this experiment. All participants were right-handed. The subjects were asked to use their right-hand index finger to press a micro-switch with closed eyes to minimize the eye movement artifacts. The EEG signals were recorded for 5 min and each participant made two recordings: one with disc electrodes and the other with TCREs. The statistical Bonferroni *t*-test was performed to compare the signal-to-noise (SNRs) of the disc and TCREs at a significance level of 1%. The results showed that the TCREs achieved a significantly higher mean SNR of 30.46 compared to the disc electrodes’ mean SNR of 7.23. Besio et al. [24] worked with epilepsy subjects to detect High-Frequency Oscillation (HFO) using TCREs. The initial experiment was performed on healthy subjects with their eyes closed, using TCREs to detect physiological alpha waves. Subsequently, recordings from epilepsy patients were obtained using conventional electrodes and TCREs. The TCREs enabled the detection of HFOs (30–500 Hz) from the scalp, which are difficult to capture using conventional disc electrodes. The results show that HFOs were detected in approximately 35.5% of the TCRE channels prior to the onset of a seizure, and among these, 78.2% were located within the seizure onset zone and irritative zone. However, some HFOs were also detected outside the onset region, indicating that TCREs improve spatial resolution, but signals are still not confined to the TCRE region.

Toole et al. [26] also worked with epilepsy patients to record the onset of seizures. Conventional electrodes and TCREs were used to record High-Frequency Activity (HFA) during seizures. The study was conducted on nine patients, of whom 8 patients had seizures during recording and one had epileptic activity. The results show that HFA detected using TCREs were more localized to the cortical regions and found in seizure onset and irritative zones. However, some HFA was found outside the electrodes’ region. This shows that TCRE can non-invasively detect epileptic activity with good resolution.

**Figure 1:**
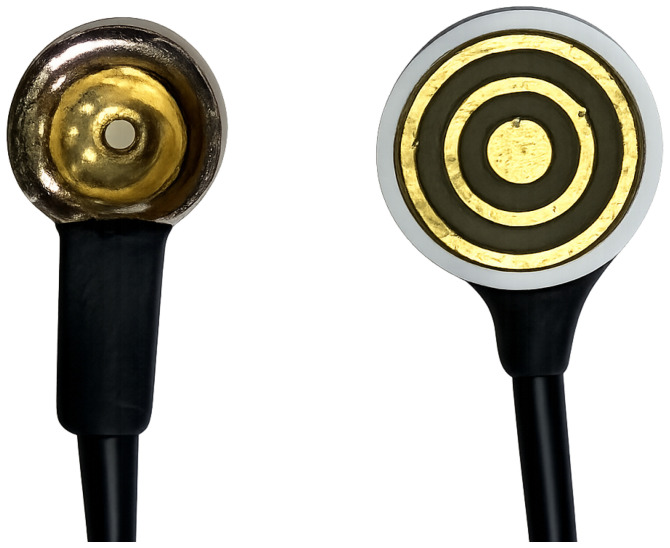
Structural difference between disc and tripolar concentric ring electrodes (TCREs).

Liu et al. [27] compared TCREs and conventional electrodes to analyze the spatial resolution of EEG signals. The visual evoked experiment was conducted to collect visual evoked potentials (VEPs) to investigate TCRE-based Laplacian sensing. In addition, TCRE-based VEPs were also collected using computer simulation. The TCRE results show a tenfold improvement in spatial resolution compared to conventional electrodes. Furthermore, TCREs were more capable of isolating human VEP activity that was not separable using conventional electrodes. These findings show that TCREs offer a great advantage for applications that require enhanced spatial selectivity in EEG-based neural signals. Alzahrani et al. [28] investigated TCREs on motor tasks such as finger movements. Their study compared conventional and TCREs. Conventional electrodes have a limited ability to distinguish the overlapped neural activities that occur in the sensorimotor cortex. To address this issue, TCREs were used to record movement-related potentials. The results showed that the TCREs achieved a higher SNR with low mutual information [29,30] and reduced the electrode reference problem. Based on the previous studies related to overt and covert speech, a common limitation is the data recorded from conventional EEG, which suffers from poor spatial resolution, low SNR, and an electrode reference problem [23,24]. Therefore, there is a need to improve the electrode design and the recording protocol. In this regard, TCREs have been used as an alternative because they provide a hardware-based estimate of the surface Laplacian (SL), which may improve spatial selectivity and reduce the influence of common activity, reference electrode effects, and signal contamination. Figure 1 represents the structural difference between the TCRE and disc electrodes.

Table 1 compares the EEG studies according to their electrode configurations and applications. To the best of the authors’ knowledge, the application of TCREs to overt and covert speech classification has not been previously investigated. Therefore, the present study evaluates whether the improved spatial selectivity provided by TCREs can enhance the discrimination of overt and covert speech-related EEG activity compared to disc electrodes.

### 1.3. Study Objectives and Contribution

The present study compares TCRE and disc electrode recordings to discriminate overt and covert speech. The objective is not to decode the linguistic content, but to determine which electrode configuration affects the separability of these conditions. The machine learning and deep learning algorithms will be used to analyze the comparison between the two electrodes’ configurations. The main contributions of this study are: (i) a comparative evaluation of TCRE and disc electrodes for overt and covert speech discrimination; and (ii) an investigation of machine learning and deep learning approaches for differentiating overt and covert speech using both electrode configurations.

## 2. Methodology

**Figure 2:**
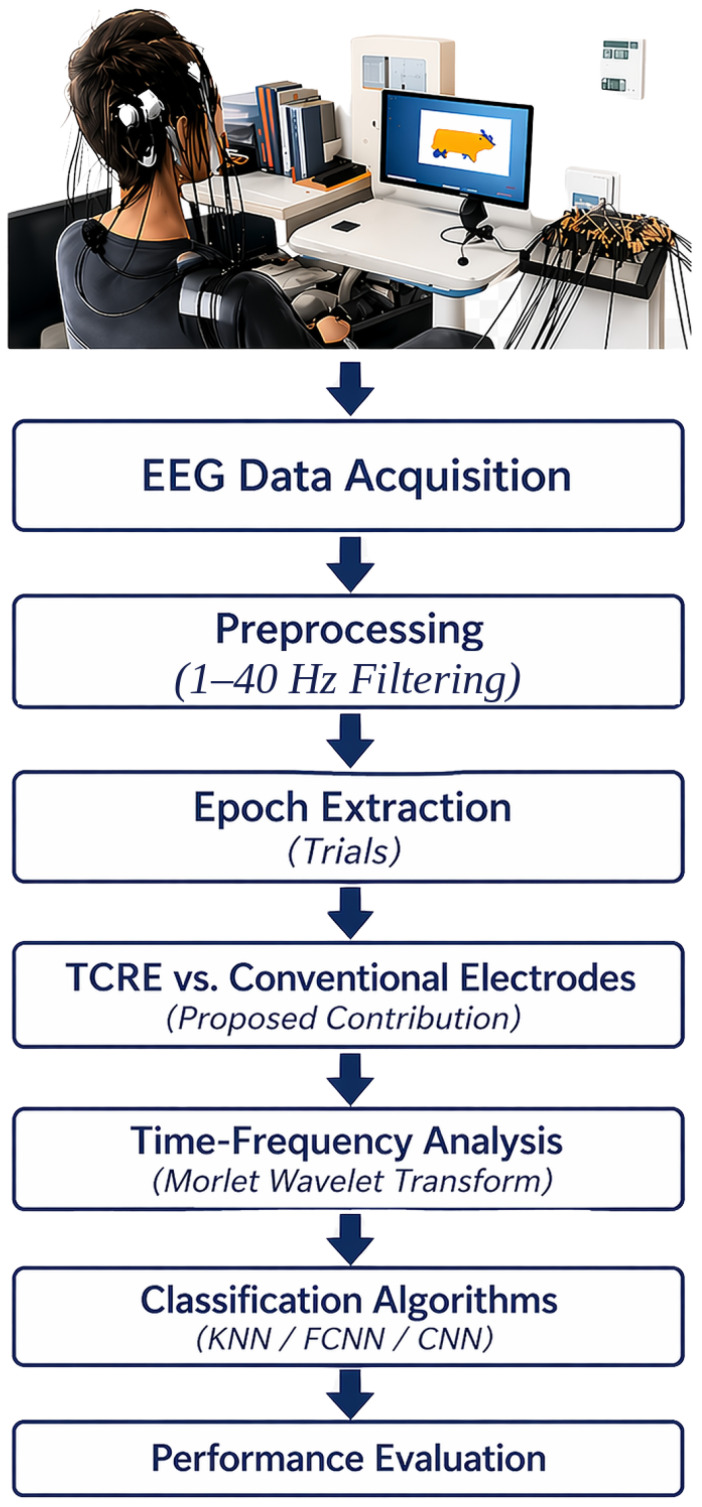
Overview of classification of TCRE and conventional electrodes EEG signal recorded using over/covert speech.

This study aims to classify disc electrodes and TCRE signals based on overt and covert speech data. Figure 2 represents the methodology of the experiment carried out in this study.

### 2.1. EEG Data Acquisition

**Figure 3:**
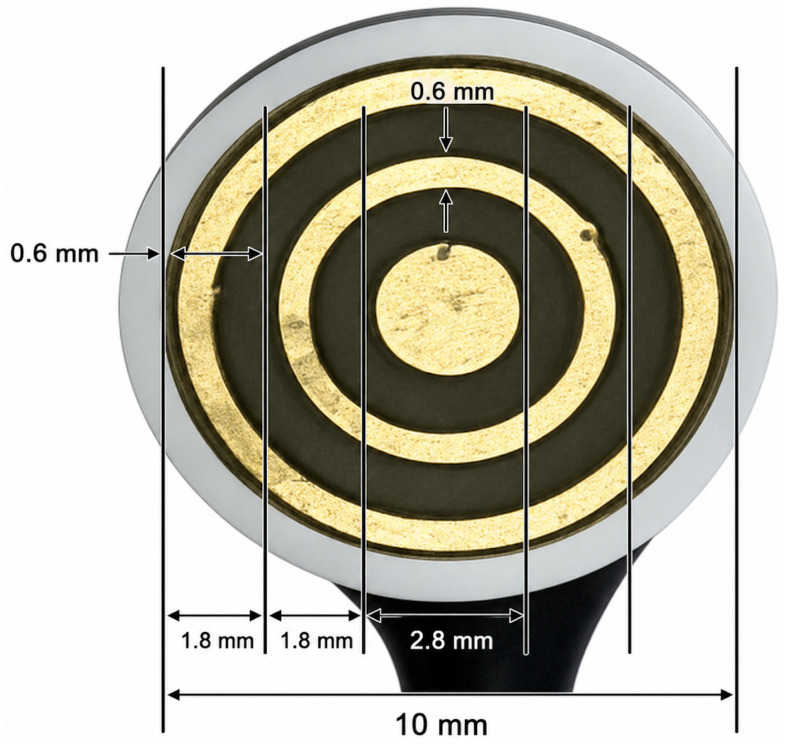
Representative geometry of a tripolar concentric ring electrode (TCRE) [32].

In this study, EEG data were recorded using TCRE and conventional disc electrodes. The participants’ EEG signals were recorded at a sampling rate of 1000 Hz during the experiment. The four electrode arrays were centered at the F3, F4, P5, and P6 scalp locations according to the international 10–20/10–5 EEG electrode placement systems [31]. The F3 and P5 locations were positioned over the left hemisphere, while F4 and P6 were positioned on the right hemisphere. The emulated disc electrodes’ signals were obtained from the outer ring of the TCREs. The reference and ground electrodes were fixed at the right mastoids. The skin to electrode impedance was maintained below 10 kΩ for both TCRE and conventional disc electrodes during recordings. The same acquisition system, electrode locations, and impedance criterion were used for both electrode types to ensure fair comparison and minimize differences arising from recording conditions. The TCRE consists of a central conductive disc surrounded by two electrically isolated concentric ring electrodes. A representative TCRE design has a diameter of 10 mm. The rings widths are 1.8 mm, central disc diameter is 2.8 mm and the insulated gap is 0.6 mm [32] as shown in Figure 3.

#### 2.1.1. Participants

Data were obtained from 16 healthy participants, consisting of 10 males and 6 females. Fourteen participants were right-handed and two were left-handed. It is observed that there is high variability between the participant data and inter-trial variability within the same participants’ EEG signals [33]. Data for this article were recorded in the Neural Rehabilitation Lab at the University of Rhode Island (URI). Before the experiment, all participants provided their consent, which was approved by the URI’s Institutional Review Board (IRB). Each participant performed two runs: overt and covert. Each run recorded 100 trials. In total, 200 trials were recorded for each participant, with overt and covert trials interspersed.

#### 2.1.2. Experimental Naming Paradigm

The participants performed both overt and covert speech tasks. During overt speech trials, participants were instructed to speak aloud the name of image shown on the computer screen as shown in Figure 2. During the covert speech, participants were instructed to imagine the corresponding words. Each stimulus was displayed for 3500 milliseconds, followed by a 2500 ms response period during which participants performed either the overt or covert task. Each participant performed two runs: overt and covert. Each run consisted of 100 trials, resulting in a total of 200 trials per participant.

### 2.2. Signal Preprocessing

Raw EEG signals contain noise and artifacts such as eye blinks, muscle activity, and body movements. To improve signal quality, a finite impulse response (FIR) band-pass filter was applied to the EEG recordings. The filter had a length of 8251 samples with cutoff frequencies of 1 Hz and 40 Hz. The lower cutoff frequency was selected to suppress slow baseline drift, DC offsets, and electrode-related artifacts, whereas the upper cutoff frequency was used to attenuate high-frequency noise, muscle artifacts, and power-line interference [34]. This preprocessing step improved the signal-to-noise ratio while preserving the EEG frequency bands relevant to speech-related neural activity.

### 2.3. Epoch Extraction

Once the preprocessing step was done, the EEG signals were segmented into epochs ranging from 0 to 2.5 s relative to stimulus onset. Each epoch represented the neural activity within a time window that followed the stimulus. No baseline correction was applied during the epoch’s extraction because the analysis focused on task-related neural activity following the stimulus response. The epoch window remained entirely within the stimulus response period. This approach minimized the boundary effects and prevent contamination from neighboring trials.

### 2.4. TCRE and Disc Electrode Separation

Data collected using TCRE and emulated disc electrode signals were recorded from the outer ring of each TCRE. The EEG signals from each electrode type were separated to evaluate their performance in overt and covert speech classification.

### 2.5. Time–Frequency Analysis

EEG signals are inherently non-stationary with spectral characteristics that vary over time. Therefore, Time-Frequency Analysis (TFR) is required to characterize the temporal evolution of neural oscillations associated with overt and covert speech tasks [35]. In this study, the Morlet wavelet transform was used because it provides a balance between temporal and frequency resolution. This makes it suitable for analyzing transient EEG activity [36]. The Morlet wavelet can be expressed as

(1) $$\psi \left(t,f\right)=e^{i2\pi ft}e^{-\frac{t^{2}}{2\sigma_{t}^{2}}}$$

where *t* denotes time, *f* is the center frequency of the wavelet, and ($\sigma_{t}$) controls the temporal width of the Gaussian envelope. The complex exponential term $e^{i2\pi ft}$ represents the oscillatory component of the wavelet, while the Gaussian term localizes the oscillation in time. This formulation enables simultaneous time and frequency localization, making the Morlet wavelet particularly suitable for analyzing non-stationary EEG signals associated with overt and covert speech.

TFR were computed using Morlet wavelets within the frequency range of 4–40 Hz. The number of wavelet cycles was set to half the analyzed frequency. This provides the adaptive trade-off between the temporal and spectral resolution across frequencies. The log-ratio baseline correction was computed as

(2) $$P_{\log}\left(t,f\right)=\log\left(\frac{P(t,f)}{P_{\mathrm{baseline}}\left(f\right)}\right)$$

where P(t,f) is the time-frequency power at time (*t*) and frequency (*f*), and $P_{\mathrm{baseline}}\left(f\right)$ is the mean spectral power calculated during the pre-stimulus baseline interval (−0.5 to 0 s). This normalization reduces the influence of inter-trial variability and emphasizes task-related changes in neural activity relative to baseline activity. The TFR were used to visualize and interpret neural activity patterns associated with overt and covert speech. No features were extracted from the TFR. Instead, the segmented EEG epoch was directly used as input to the machine learning algorithms.

### 2.6. Classification Algorithms

Classification algorithms were used to evaluate the effectiveness of the TCRE and disc electrodes for overt and covert classification. Three classification algorithms, KNN, FCNN, and CNN, were evaluated separately. KNN was chosen as a simple non-parametric classifier. It has been widely used in EEG-based BCI studies because of its computational efficiency and ability to perform effectively on relatively small datasets [1,37]. FCNN was selected because of its ability to learn complex nonlinear relationships within EEG signals through multiple hidden layers. It can enable the extraction of discriminative patterns associated with neural activity [38]. CNN was used because of its capability to automatically learn hierarchical feature representations and capture local temporal characteristics of EEG signals without requiring the feature extraction [39]. By employing both machine learning approach (KNN) and deep learning approaches (FCNN and CNN), a comparison of classification performance was performed between TCRE and disc EEG for overt and covert classification.

#### 2.6.1. K-Nearest Neighbors (KNN)

The KNN classifier was used as a non-parametric supervised learning algorithm for overt and covert speech classification. Prior to classification, each EEG epoch was reshaped into a feature vector by concatenating the channel and temporal samples. The similarity between a test sample and the training samples was quantified using the Euclidean distance

(3) $$d\left(x_{i},x_{j}\right)=\sqrt{\sum_{m=1}^{n}{\left(x_{im}-x_{jm}\right)}^{2}}$$

where $x_{i}$ and $x_{j}$ represent two EEG feature vectors, *n* is the total number of features, and $d(x_{i},x_{j})$ is the Euclidean distance between them. The class label of a test sample is determined by majority voting among its *k* nearest neighbors. To optimize the classifier, the number of neighbors was varied from k=1 to k=9, and the optimal value of *k* was selected using fivefold cross-validation on the training data. The final KNN model was trained using the selected value of *k* and subsequently evaluated on the independent testing dataset.

#### 2.6.2. Fully Connected Neural Network (FCNN)

The FCNN classifier was implemented using a multilayer perceptron (MLP) architecture. Prior to classification, each EEG epoch was reshaped into a feature vector by concatenating the channel and temporal samples. The output of each hidden layer is computed as

(4) h=f(Wx+b),

where x is the input feature vector, W is the weight matrix, b is the bias vector, f(·) denotes the activation function, and h represents the hidden-layer output. In this study, the rectified linear unit (ReLU) activation function was used together with the Adam optimizer. The different hidden layer configurations were evaluated: (20,5), (20,10), and (30,10,10), where each value represents the number of neurons in a hidden layer. The maximum number of training iterations was varied among 10, 20, 30, and 40 epochs. The optimal combination of hidden layer architecture and training iterations was selected using fivefold cross-validation on the training dataset. The final FCNN model was then trained using the selected hyperparameters and evaluated on the independent testing dataset.

#### 2.6.3. Convolutional Neural Network (CNN)

Unlike conventional machine learning algorithms that require manual feature extraction, CNNs can learn hierarchical feature representations directly from the input data. The convolution operation can be expressed as

(5) $$y_{i}=\sum_{j=0}^{k-1}w_{j}x_{i+j}+b$$

where *x* represents the input EEG signal, *w* denotes the convolution kernel weights, *k* is the kernel size, *b* is the bias term, and $y_{i}$ is the output feature map at location *i*.

The CNN classifier was implemented using a convolutional neural network architecture to automatically learn discriminative features from the EEG signals. Several convolutional and fully connected network configurations were investigated to identify an appropriate model complexity for overt and covert speech classification. The models were trained and the optimal architecture was selected using fivefold cross-validation on the training dataset. The final CNN model was subsequently evaluated on the independent testing dataset.

### 2.7. Performance Evaluation

The classification procedure was performed in a subject-dependent manner. For each participant, the available trials were first divided into 80% training data and 20% held-out testing data. The testing data were not used during model training or validation. A fivefold cross-validation procedure was then applied only to the 80% training data to train and validate the classification models to reduce the risk of overfitting [40].

After completion of the training process, the final model was evaluated using the independent 20% testing data. This procedure was repeated separately for each participant. Therefore, trials from different participants were not combined during training or testing phase. This separation ensured that the testing data remained unseen during training and cross-validation, reducing the possibility of data leakage between the training and testing stages. The final performance of the models was evaluated using classification accuracy and confusion matrices.

## 3. Results and Discussion

This section presents the results of EEG based overt and covert speech using TCRE and disc electrodes.

### 3.1. Overall Performance Comparison

Table 2 summarizes the participant-wise classification accuracies obtained using TCRE and conventional disc electrodes. The results show substantial variability among participants. Overall, TCREs achieved higher mean accuracies than conventional disc electrodes for all three classification algorithms. The mean accuracies obtained using TCREs were 73.44±16.71%, 60.16±14.70%, and 72.22±19.61% for KNN, FCNN, and CNN, respectively. On the other hand, disc electrodes achieved accuracies of 61.25±15.99%, 50.63±9.56%, and 67.67±15.47% for KNN, FCNN, and CNN, respectively.

Among the classification algorithms, KNN achieved the highest average classification accuracy. Several participants, including P05, P07, P11, and P16, achieved accuracies above 90% when using TCREs. In contrast, P01, P09, and P10 achieved lower accuracies suggesting that overt and covert speech patterns are more difficult to distinguish for some participants. These findings suggest that both electrode type and classification algorithm influence the discrimination of overt and covert EEG signals. This can be attributed to inter subject variability in neural activity, attention, task, and EEG signal quality [1,41,42].

### 3.2. Comparison of Classification Algorithms Using TCRE and Disc Electrodes

**Figure 4:**
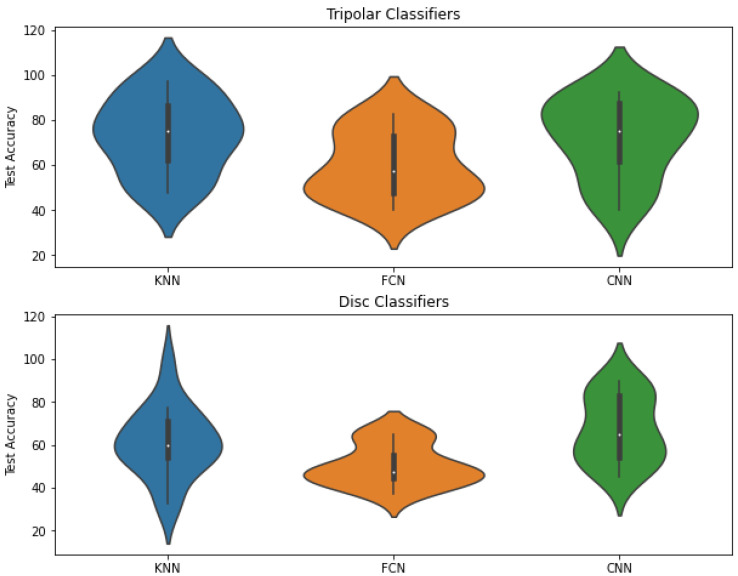
Distribution of classification accuracies for KNN, FCNN, and CNN across all participants using TCRE and conventional disc electrodes.

Figure 4 shows the distribution of classification accuracies for KNN, FCNN, and CNN among all participants using both TCRE and disc electrodes, while Table 2 reports the numerical accuracy values for each participant. Figure 4 provides an overall view of how the accuracies are distributed among the participants. For the TCRE EEG, both KNN and CNN show a larger concentration of accuracies in the upper range compared with the corresponding disc electrode results. On the other hand, FCNN exhibits a wider spread of lower accuracy values, which is consistent with its lower mean accuracy. The figure also demonstrates that classification performance varies among participants regardless of the classification algorithm. Overall, the distributions indicate that TCRE recordings generally produce higher classification accuracies than conventional disc electrodes and provide more consistent performance across participants.

### 3.3. Statistical Comparison Between TCRE and Disc Electrodes

The classification accuracies obtained using TCRE and conventional disc electrodes were significantly different. The Wilcoxon signed-rank test was performed on the participant-wise accuracies. The Wilcoxon test was selected because of the small sample size (n=16) of the dataset [43]. The statistical significance was determined at α=0.05. Table 3 summarizes the Wilcoxon signed-rank test results comparing TCRE and disc electrodes. A significant difference was observed for TCREs when using KNN (p=0.0097) and FCNN (p=0.0199). On the other hand, there was no significant difference observed between TCREs and conventional disc electrodes using the CNN algorithm (p=0.2979). These results suggest that TCREs improve the classification performance of the KNN and FCNN classification algorithms.

### 3.4. Statistical Comparison Between Classification Algorithms

To evaluate the classification algorithms’ performance, Wilcoxon signed-rank tests were performed on participant-wise TCRE accuracies. The Wilcoxon test was selected because the sample size was relatively small (n=16) and the accuracies were paired within the same participants. The statistical significance was assessed at α=0.05. Table 4 summarizes the statistical comparison among KNN, FCNN, and CNN. The results showed that KNN achieved significantly higher classification accuracy than FCNN (p=0.0082) and no statistically significant differences were observed between KNN and CNN (p=0.4776). Nonetheless, CNN achieved significantly higher classification accuracy than FCNN (p=0.0478). These results indicate that KNN and CNN produced comparable performance. However, FCNN exhibited relatively lower classification performance.

CNNs are widely used for EEG classification due to their ability to learn complex spatiotemporal representations [1]. However, the present study did not find any significant difference between the performance of KNN and CNN (p=0.4776). One possible explanation is the limited size of the dataset, which consisted of 16 participants and 200 trials per participant. Deep learning models generally require larger datasets to learn robust feature representations to avoid overfitting [1,38]. In contrast, KNN is a non-parametric distance-based classifier that can perform effectively on the smaller datasets by exploiting local similarities between samples [37]. Furthermore, the classification algorithms were applied directly on the EEG epochs without feature extraction. Therefore, KNN and CNN algorithms achieve comparable performance.

### 3.5. Classifier Performance Analysis

**Figure 5:**
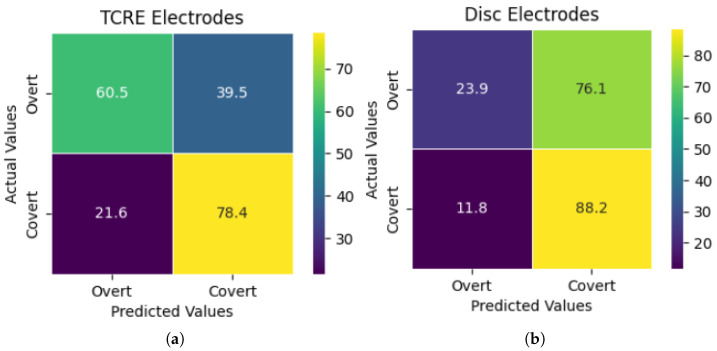
Confusion matrix of TCRE and disc electrodes using KNN: (**a**) 10 mm TCRE; (**b**) regular disc electrodes.

**Figure 6:**
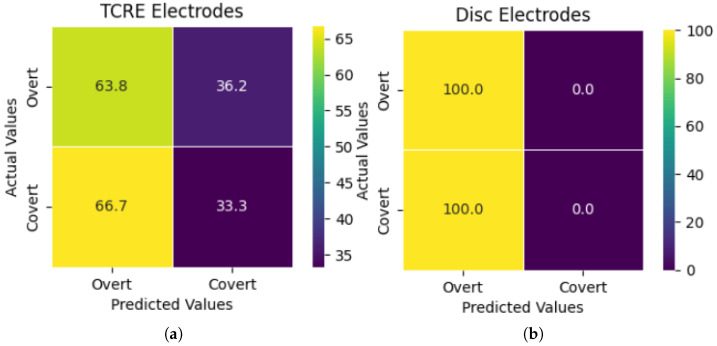
Confusion matrix of TCRE and disc electrodes using FCNN: (**a**) 10 mm TCRE; (**b**) regular disc electrodes.

**Figure 7:**
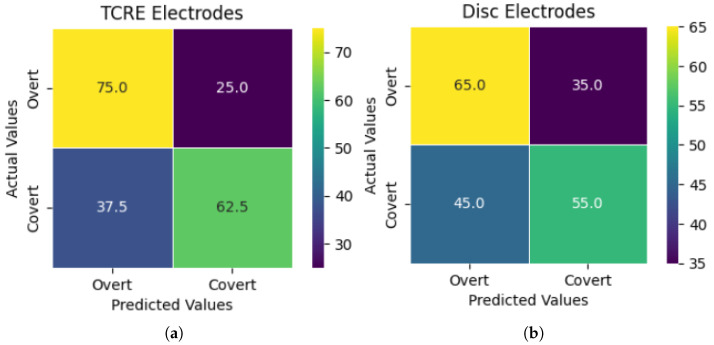
Confusion matrix of TCRE and disc electrodes using CNN: (**a**) 10 mm TCRE; (**b**) regular disc electrodes.

The classification algorithms, KNN, FCNN, and CNN, were evaluated using confusion matrices. The confusion matrices are shown in Figure 5, Figure 6 and Figure 7. It is observed that KNN consistently achieved higher results compared to CNN and FCNN. KNN performs effectively because it exploits local similarities between EEG samples without requiring large amounts of EEG data. CNN also demonstrates a strong performance, especially using TCRE EEG signals. In contrast, FCNN presents a relatively lower performance. This represents its limited ability to capture spatiotemporal EEG patterns. These results suggest the importance of selecting optimal classification algorithms for EEG-based overt/covert differentiation.

### 3.6. Epoch Segmentation and Analysis

**Figure 8:**
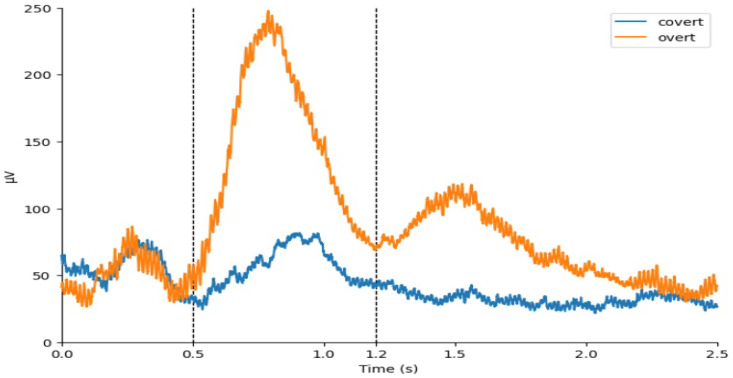
Participant 11—Analysis of peak epoch between (0.5–1.2) Vs. remaining non-peak epoch.

In this section, we examine the time duration of the epoch (0–2.5 s). For analysis purposes, a participant was taken from the overt and covert dataset to investigate the variation in the epoch over time. Figure 8 represents the overt and covert EEG signals of Participant 11. Each epoch consits of two parts: peak and non-peak. Peak is the amplitude of signals over the time when the participant speaks aloud (overt) or silently imagines (covert) the name of the image shown on the computer to record the brain activity. Non-peak refers to the neural activity recorded before and after the peak activity. Each epoch represents the average response of 200 trials that were collected for a single participant.

The peak interval (0.5–1.2 s) corresponds to the period where the difference between overt and covert EEG responses is very clear. During this interval, speech neural activity is expected to be strongest, resulting in greater separability between the two classes. The peak interval achieved a mean classification accuracy of 86.25%, whereas the non-peak epoch achieved 83.5%. Although the difference is small, the results suggest that the peak interval contains slightly more discriminative information that can be used to distinguish overt and covert speech.

**Figure 9:**
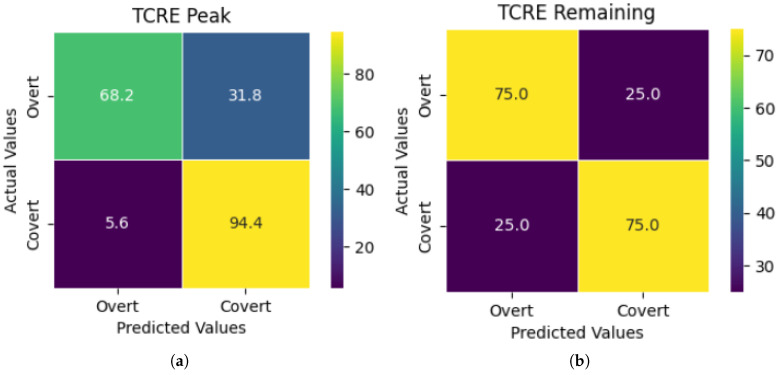
Confusion matrix of epochs at peak vs. non-peak. (**a**) Peak of the epoch; (**b**) non-peak of the epoch.

Figure 9 exhibits the confusion matrix for classification algorithm KNN on data from participant 11. The overt data are correctly classified at a rate of 68.2% while experiencing a misclassification rate of 31.8%. In contrast, covert data are correctly classified with an accuracy of 94.4%, with a misclassification rate of 5.6%. On the other hand, at the non-peak intervals of the epoch, overt and covert runs are correctly classified at a rate of 75%, with a misclassifcation rate of 25% for both overt and covert runs.

### 3.7. Advantages and Limitations of TCRE

The present study demonstrated that TCREs achieved higher mean classification accuracies than conventional disc electrodes. These findings are consistent with previous investigations that reported improved spatial selectivity and signal quality using TCREs [25] Earlier studies demonstrated the effectiveness of TCREs for applications such as seizure detection, motor-task classification, and visual evoked potential analysis. The results of the present study extend these findings by demonstrating the potential of TCREs for overt and covert speech classification.

TCREs offer several advantages over conventional disc electrodes for speech-related EEG recordings. The concentric ring configuration provides improved spatial resolution and enhanced localization of neural activity compared with conventional EEG [25,27].

Compared with other neural recording modalities used for speech, TCRE-based EEG offers several practical advantages. ECoG can provide higher spatial resolution and decoding performance; however, it requires invasive surgical implantation of electrodes. MEG and fMRI provide high-quality neural measurements but require expensive and non-portable instrumentation. In contrast, TCRE-based EEGs are non-invasive, portable, and relatively low cost while providing improved spatial selectivity compared with conventional disc-electrode EEGs.

The proposed TCRE-based EEG framework has potential applications in biomedical signal analysis and brain–computer interface (BCI) systems for both overt and covert speech classification. Covert speech decoding is particularly important for individuals who are unable to communicate through normal speech due to neurological disorders, motor impairments, or severe paralysis. In such cases, covert speech-based BCIs may provide an alternative communication pathway by translating internally generated speech intentions into commands or text [44]. The TCRE-based EEG recordings may support applications in neurorehabilitation, assistive technologies, and human–computer interaction by improving the detection of speech-related neural activity.

Despite these advantages, several limitations should be acknowledged. TCRE recordings require specialized electrode hardware and amplification systems, which may limit their widespread adoption compared with conventional EEG systems. In addition, the present study was conducted using a relatively small dataset consisting of 16 participants. Although model selection was performed using fivefold cross-validation and an independent testing dataset, a larger participant dataset would provide a more comprehensive evaluation of the proposed framework. Furthermore, complete demographic information, including age was not available in the dataset, preventing a detailed assessment of the influence of participant characteristics on classification performance. Future studies should investigate larger and more diverse participant populations.

## 4. Conclusions

This study compared TCRE and disc electrode EEG signals for classifying overt and covert speech. The results showed that the TCRE recordings produced higher classification accuracies than disc electrode recordings for most classification algorithms. Among the classifiers evaluated, KNN achieved the highest average accuracy using TCRE signals, followed by CNN. FCNN achieved a lower average accuracy for both TCRE and disc electrode signals. The epoch segment analysis showed that the 0.5–1.2 s interval provided better separability between overt and covert speech. Overall, the results suggest that TCREs are useful for non-invasive BCI applications.

Future studies should use a larger and more diverse participant dataset and examine more complex speech tasks, such as word-level and vocabulary based decoding. 

## Figures and Tables

**Table 1:** Summary of EEG studies: A checkmark (✓) indicates the electrode type used in each study (Disc or TCRE). shaded cells represent no prior work done on the speech decoding using TCRE.

Reference	Electrode Type		Application	Key Finding
	Disc	TCRE		
Hossain et al. [16]	✓	–	Speech Decoding	85.65% (letters)
				83.65% (digits)
Jiang et al. [17]	✓	–	Speech Decoding	34.7% covert speech
Milyani and Attar [19]	✓	–	Speech Decoding	82.4% (Transformer)
Alharbi and Alotaibi [20]	✓	–	Speech Decoding	75.2%
Abdulghani et al. [21]	✓	–	Speech Decoding	92.50%
Besio et al. [25]	–	✓	Motor Task	SNR improved from 7.23 to 30.46
Besio et al. [24]	–	✓	HFO Detection	35.5% onset seizure detection
Toole et al. [26]	–	✓	HFA Localization	HFA localized in onset & irritative zone
Liu et al. [27]	–	✓	Spatial Resolution	10× improvement
Alzahrani et al. [28]	–	✓	Motor Task	Improved finger movement discrimination

**Table 2:** Classification accuracies (%) obtained using TCRE and disc electrodes for all participants. Final row shows the mean and standard deviation across all participants.

Participants	TCRE (%)			Disc (%)		
	KNN	FCNN	CNN	KNN	FCNN	CNN
P01	55.0	45.0	45.0	32.5	47.5	50.0
P02	85.0	72.5	75.0	55.0	45.0	77.5
P03	75.0	75.0	75.5	60.0	37.5	68.0
P04	67.5	80.0	67.5	35.0	52.5	65.0
P05	90.0	82.5	87.5	65.0	65.0	82.5
P06	50.0	57.49	52.5	62.5	65.0	60.0
P07	92.5	57.49	92.5	70.0	50.0	85.0
P08	77.5	50.0	87.5	75.0	47.5	85.0
P09	47.5	50.0	40.0	52.5	40.0	65.0
P10	50.0	45.0	50.0	55.0	45.0	45.0
P11	97.5	82.5	85.5	77.5	65.0	88.75
P12	65.0	60.0	65.0	55.0	65.0	55.0
P13	77.5	70.0	70.0	52.5	52.5	52.5
P14	75.0	40.0	92.0	60.0	42.5	50.0
P15	72.5	47.5	80.0	75.0	47.5	90.0
P16	97.5	47.5	90.0	97.5	42.5	57.49
Mean ± SD	73.44 ± 16.71	60.16 ± 14.70	72.22 ± 19.61	61.25 ± 15.99	50.63 ± 9.56	67.67 ± 15.47

**Table 3:** Statistical comparison of TCREs and disc electrodes using Wilcoxon signed-rank test.

Classification Algorithm	*p*-Value
KNN	0.0097
FCNN	0.0199
CNN	0.2979

**Table 4:** Comparison of classification algorithms using Wilcoxon signed-rank test.

Comparison	*p*-Value
KNN vs. FCNN	0.0082
KNN vs. CNN	0.4776
CNN vs. FCNN	0.0478

## Data Availability

The data used in this study are not publicly available due to institutional restrictions. Data may be made available from the corresponding author with appropriate institutional approval.

## Abbreviations

The abbreviations used in this manuscript are these:

BCI	Brain Computer Interface
EEG	Electroencephalography
ECoG	Electrocorticography
EMG	Electromyography
fMRI	Functional Magnetic Resonance Imaging
MEG	Magnetoencephalography
TCRE	Tripolar Concentric Ring Electrode
KNN	K-Nearest Neighbors
FCNN	Fully Connected Neural Network
CNN	Convolutional Neural Network
SNR	Signal to Noise Ratio
HFO	High Frequency Oscillation
